# Genetic control of juvenile growth and botanical architecture in an ornamental woody plant, *Prunus mume *Sieb. et Zucc. as revealed by a high-density linkage map

**DOI:** 10.1186/1471-2156-15-S1-S1

**Published:** 2014-06-20

**Authors:** Lidan Sun, Yaqun Wang, Xiaolan Yan, Tangren Cheng, Kaifeng Ma, Weiru Yang, Huitang Pan, Chengfei Zheng, Xuli Zhu, Jia Wang, Rongling Wu, Qixiang Zhang

**Affiliations:** 1 Beijing Key Laboratory of Ornamental Plants Germplasm Innovation and Molecular Breeding, National Engineering Research Center for Floriculture, College of Landscape Architecture, Beijing Forestry University, Beijing 100083, China; 2 Center for Statistical Genetics, Pennsylvania State University, Hershey, PA 17033, USA; 3 Mei Research Center of China, Wuhan 430074, China; 4 Center for Computational Biology, College of Biological Science and Technology, Beijing Forestry University, Beijing 100083, China

**Keywords:** growth, linkage map, Mei, morphology, QTL

## Abstract

Mei, *Prunus mume *Sieb. et Zucc., is an ornamental plant popular in East Asia and, as an important member of genus *Prunus*, has played a pivotal role in systematic studies of the Rosaceae. However, the genetic architecture of botanical traits in this species remains elusive. This paper represents the first genome-wide mapping study of quantitative trait loci (QTLs) that affect stem growth and form, leaf morphology and leaf anatomy in an intraspecific cross derived from two different mei cultivars. Genetic mapping based on a high-density linkage map constricted from 120 SSRs and 1,484 SNPs led to the detection of multiple QTLs for each trait, some of which exert pleiotropic effects on correlative traits. Each QTL explains 3-12% of the phenotypic variance. Several leaf size traits were found to share common QTLs, whereas growth-related traits and plant form traits might be controlled by a different set of QTLs. Our findings provide unique insights into the genetic control of tree growth and architecture in mei and help to develop an efficient breeding program for selecting superior mei cultivars.

## Introduction

Mei, *Prunus mume *Sieb. et Zucc., a species of genus *Prunus*, is a popular ornamental plant, originated in Southwest China [1] and widely cultivated in the entire East Asia [1,2]. Its cold hardiness by blooming in winter or early spring, plus its many prominent ornamental features, such as colorful corollas, varying flower forms, and pleasant fragrance, have made it a symbol of spirit in Chinese culture, favorably praised by litterateurs and ordinary people [1]. Fruits of mei have also been used as raw material to make Chinese herbal medicine beneficial for human health [2]. As an important member of sub-family Prunoideae, mei is a key step towards constructing a phylogenetic tree for family Rosaceae, thought to play a pivotal role in understanding the evolution of woody plants [3].

Because of its significant value in biological research and practical cultivation, mei has received increasing attention during the past years [3-8]. Fang et al. [4] developed a set of molecular markers, such as AFLP and SNP, to investigate the genetic relatedness and diversity of 50 cultivars of fruiting mei from China and Japan. Similar work using AFLP markers was conducted by Yang et al. [5] to analyze the genetic diversity of ornamental mei and compare it with that of other related species. Li et al. [6] developed more informative multiallelic microsatellite markers, i.e., simple sequence repeats (SSRs), from 20 mei plants, particularly useful to study the genetic structure of natural populations in mei. Using two mei cultivars, Fenban and Kouzi Yudie, and five segregating progeny randomly chosen from the F_1 _intraspecific hybrid family of these two cultivars, Sun et al. [7] performed the genome-wide characterization of SSRs in the mei genome and construct a first genetic linkage map of mei using 144 SSR markers. Despite these progresses in mei genetic studies, almost nothing is known about the genetic control of its botanical traits of ornamental and biological value.

More recently, with the advent and widespread application of next-generation technologies, the genetic studies of mei have entered a new era in which multiscale data collected at the molecular, cell and organ levels enables geneticists to characterize the genetic architecture of complex phenotypes and construct a genotype to phenotype map for mei. Right after genomes of apple and strawberry, both belonging to Rosaceae, have been sequenced [9,10], Zhang et al. [3] have for the first time sequenced the mei genome, providing an impetus for studying the comparative genomics of Rosaceae and mapping important genes that contribute to mei traits. Based on the mei reference genome, Sun et al. [8] were able to identify hundreds of thousands of SNPs for cultivars Fenban and Kouzi Yudie. The F_1 _family of these two cultivars was genotyped for a couple of thousands of SNPs. By adding these segregating SNPs to the SSR linkage map, previously reported in Sun et al. [7], a high-density genetic map for mei has been generated.

In this article, we report on the detection of quantitative trait loci (QTLs) that affect stem growth, stem form and leaf morphological traits in the juvenile seedlings of mei using a segregating F1 family derived from cultivars Fenban and Kouzi Yudie [7]. Early growth and its morphological components, such as leaf size, in the first year of growth in the field are important traits associated with the ability of mei to build itself to tolerate and resist to adverse conditions, particularly low temperature and drought in early spring. Our high-density genetic map allows the genome-wide mapping and identification of QTLs responsible for the early performance of mei in the field. Results from QTL mapping are not only useful for marker-assisted selection and breeding of rigorous growth traits in mei, but also help to explore the commonality of genetic control for growth traits in Rosaceae through comparing with QTL discoveries in other species such as apple and strawberry.

## Results

Unlike an inbred line homozygous for all loci, an outcrossing line is complex in genetic composition, in which some loci are homozygous whereas the others are heterozygous [11]. Thus, the F_1 _cross of two outcrossing parents, like Fenban and Kouzi Yudie, may generate a segregating progeny as long as one parent is heterozygous for some loci. There are four possible types of segregating markers for an outcrossing family [12,13]: (1) multi (3 or 4)-allelic intercross markers, (2) bi-allelic intercross markers, (3) testcross markers that are heterozygous for one parent but homozygous for the other, and (4) testcross markers that are opposite to (3). These marker types produce four, three, two and two distinguishable genotypes in the progeny, respectively. Statistical models implemented with different numbers of effect parameters are used to identify QTLs from these markers [14].

**Figure 1:**
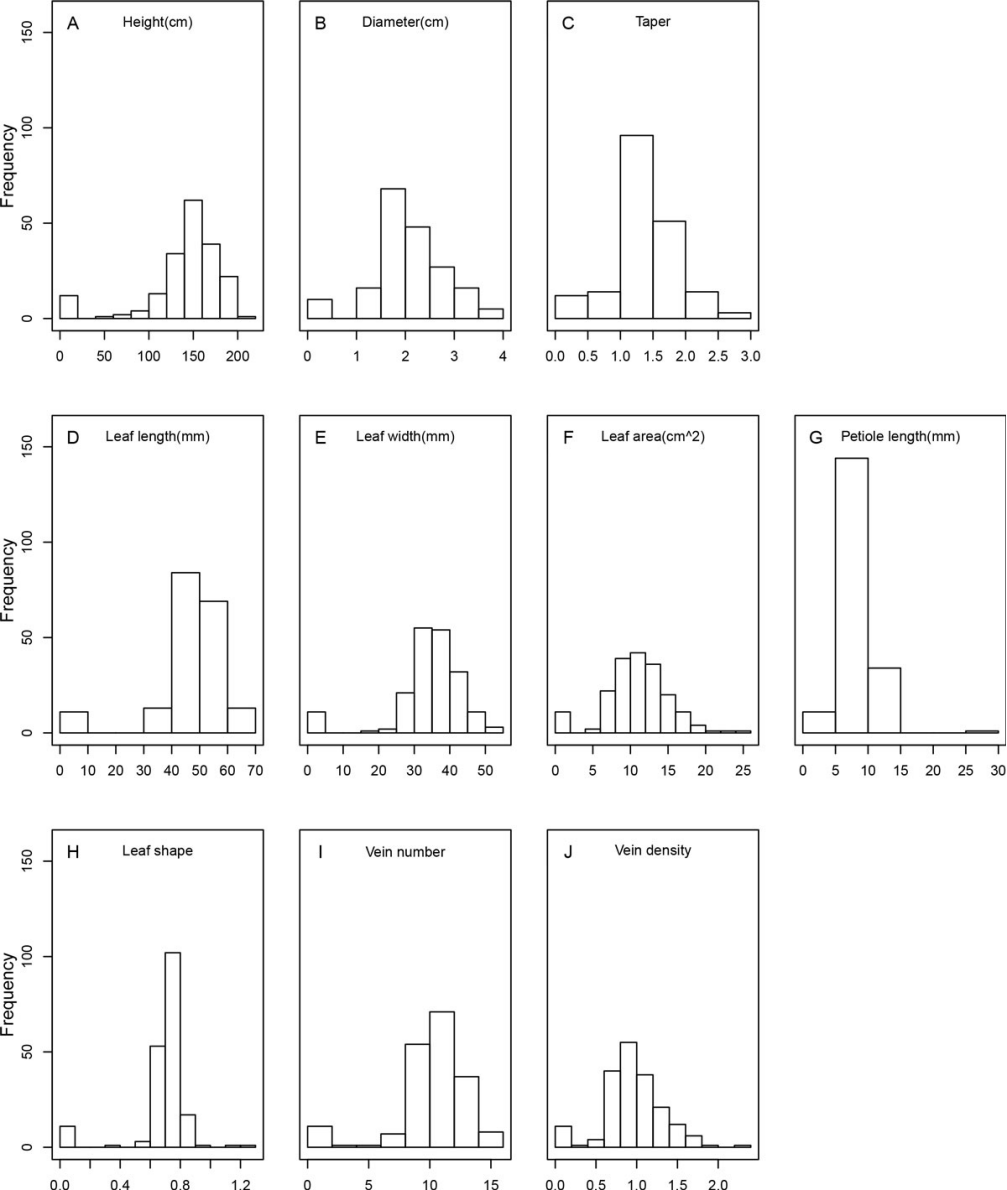
**Histograms of stem, leaf morphology and leaf anatomy traits in an F_1 _full-sib family derived from two mei cultivars, *P. mume *"Fenban" (BJFU1210120013) and *P. mume *"Kouzi Yudie" (BJFU1210120022)**.

**Figure 2:**
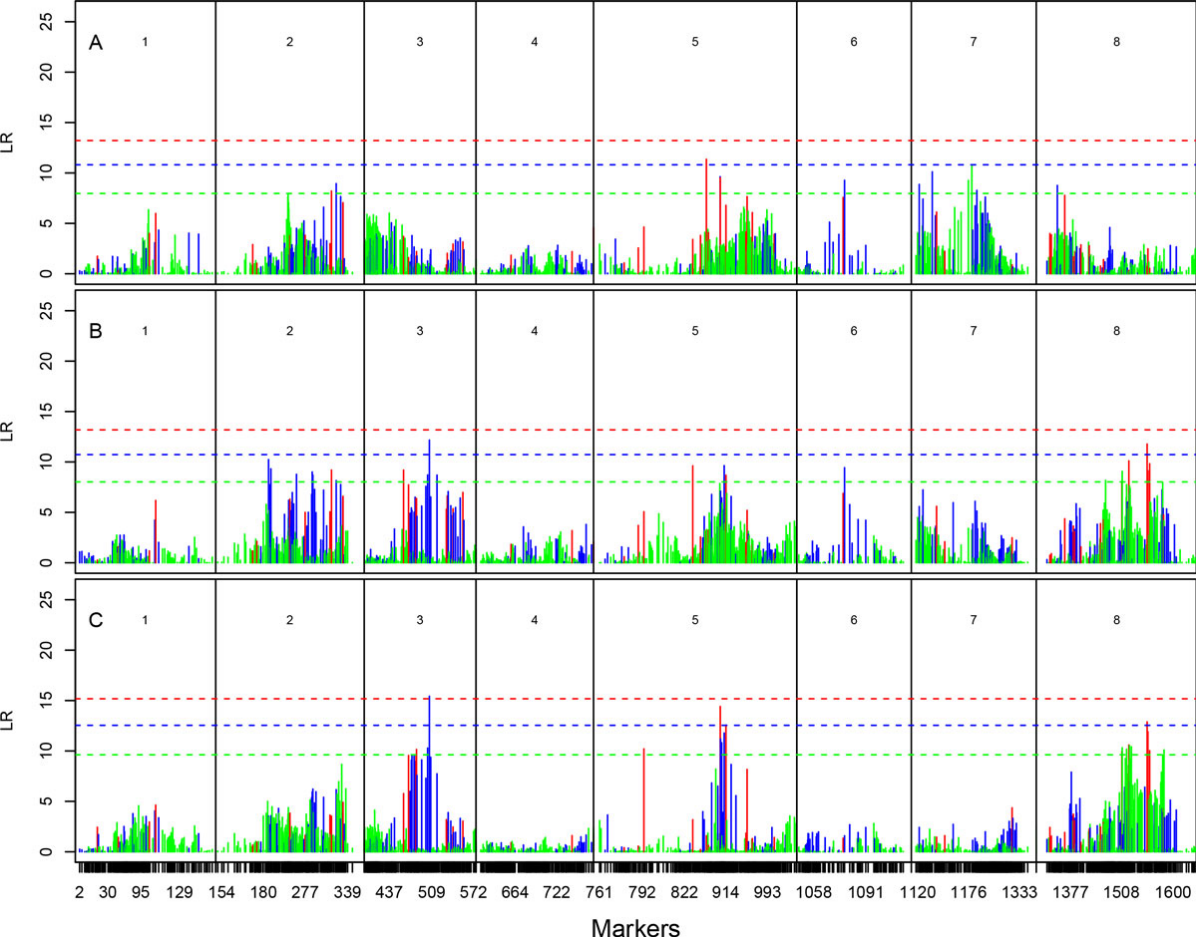
**Log-likelihood-ratio (LR) profiles of testing the genomic distribution of QTLs throughout eight linkage groups for main stem traits, stem height (A), stem diameter (B), and stem taper (C) in the first growing season of mei F_1 _hybrids grown in the field**. The positions of markers are indicated as ticks on the x-axis. Multiallelic intercross markers (with four genotypes), biallelic intercross markers (with three genotypes) and testcross markers (with two genotypes) are shown in red, blue, and green, respectively. The horizontal lines are the genome-wide critical thresholds at the 5% significance level determined through the FDR adjustment.

**Figure 3:**
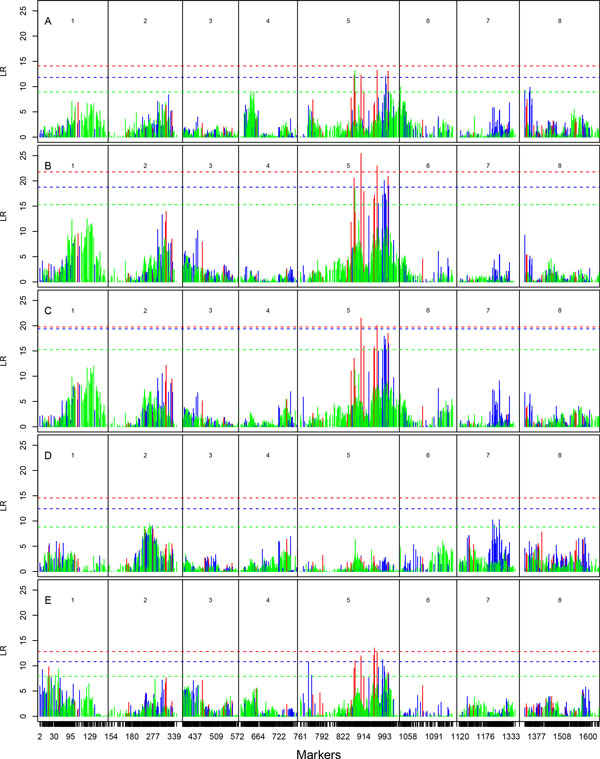
**Log-likelihood-ratio (LR) profiles of testing the genomic distribution of QTLs throughout eight linkage groups for leaf morphological traits, leafblade length (A), leaf width (B), leaf area (C), petiole length (D), and leaf shape (E) in the first growing season in the field**. The positions of markers are indicated as ticks on the x-axis. Multiallelic intercross markers (with four genotypes), biallelic intercross markers (with three genotypes) and testcross markers (with two genotypes) are shown in red, blue, and green, respectively. The horizontal lines are the genome-wide critical thresholds at the 5% significance level determined through the FDR adjustment.

**Figure 4:**
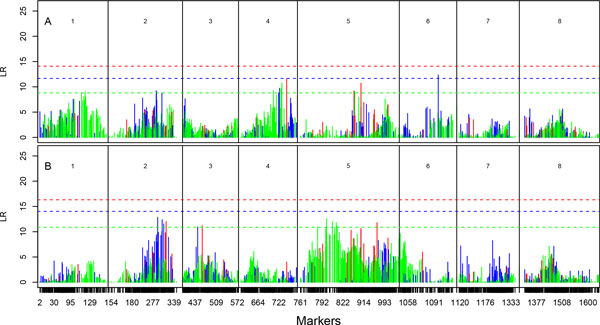
**Log-likelihood-ratio (LR) profiles of testing the genomic distribution of QTLs throughout eight linkage groups for leaf anatomical traits, the number of veins (A) and density of veins (B) in the first growing season in the field**. The positions of markers are indicated as ticks on the x-axis. Multiallelic intercross markers (with four genotypes), biallelic intercross markers (with three genotypes) and testcross markers (with two genotypes) are shown in red, blue, and green, respectively. The horizontal lines are the genome-wide critical thresholds at the 5% significance level determined through the FDR adjustment.

The juvenile phenotypic traits studied in the mapping population of mei are classified into three categories: (1) stem growth and form, described by stem height, stem diameter, and stem slenderness (measured as diameter/height ratio), (2) leaf morphology, including leaf length, length width, single leaf area, petiole length, and leaf shape (measured as length width/length ratio), and leaf structure, including the number of veins per leaf and the number of veins per unit area of a leaf (vein density). Figure 1 illustrates the histograms of each trait in the mapping population, showing an approximate normal distribution and pronounced variability. Each trait was associated with individual markers by a maximum likelihood approach. Plots of log-likelihood ratios for each trait over linkage groups are given in Figures 2, 3, 4, in which the genomic distribution of significant QTLs is shown. We did not detect many significant QTLs for stem growth, only with two for height growth jointly accounting for 7% of the phenotypic variation and three for diameter growth explaining 16% of the phenotypic variation together (Table 1; Figure 2). One diameter QTL on linkage group 8 is an intercross type, acting in an overdominant manner (d/a = 15).

**Table 1:** Additive (a) and dominant genetic effects (d) of significant QTLs, and the proportions of phenotypic variance (R^2^) explained by each of these QTLs, associated with stem growth and form, leaf morphology and leaf anatomy in an F_1 _mapping population of mei.

				Effect			
					
Trait	Marker	Linkage Group	No. Genotypes	a_1_	a_2_	d	R^2^
**Stem Growth and Form**							
Height	PMSNP01036	7	2	5.88	-	-	0.03
	PMSNP01033	7	2	6.88	-	-	0.04
Diameter	PMSNP00162	8	2	0.12	-	-	0.02
	PMSNP00095	8	2	0.13	-	-	0.03
	PMSNP00545	3	3	0.02	-	0.30	0.11
Stem Taper	PMSNP00095	8	2	0.12	-	-	0.03
	PMSNP00082	8	2	0.11	-	-	0.03
	PMSNP00071	8	2	0.13	-	-	0.03
	PMSNP00068	8	2	0.11	-	-	0.03
	PMSNP00021	3	3	0.14	-	-	0.03
**Leaf Morphology**							
Leaf Length	PMSNP01203	4	2	1.45	-	-	0.03
	PMSNP00307	5	2	1.76	-	-	0.04
	PMSNP00457	5	3	2.15	-	1.23	0.10
	PMSNP01407	6	2	1.52	-	-	0.03
Leaf Width	PMSSR0620	5	4	1.00	1.96	0.04	0.08
	PMSSR0358	5	4	1.27	1.74	0.32	0.09
	PMSNP00349	5	3	3.07	-	0.59	0.15
	PMSNP00453	5	3	2.74	-	0.44	0.14
	PMSNP00470	5	3	-2.79	-	-0.11	0.13
Leaf Area	PMSSR0620	5	4	-0.46	-1.06	0.06	0.08
	PMSSR0358	5	4	0.72	0.92	0.10	0.07
Leaf Petiole	PMSNP00815	2	2	0.54	-	-	0.03
	PMSNP00818	2	2	0.51	-	-	0.03
	PMSNP00821	2	2	0.51	-	-	0.03
Lea Shape	PMSSR0128	5	4	0.015	0.011	0.010	0.03
	PMSNP01299	1	2	0.017	-	-	0.02
	PMSNP01309	1	2	0.019	-	-	0.03
	PMSNP00463	5	2	0.018	-	-	0.02
	PMSNP00349	5	3	0.027	-	0.013	0.07
	PMSNP00448	5	3	-0.027	-	0.010	0.06
**Leaf Anatomy**							
Vein Number	PMSNP00307	5	2	-0.43	-	-	0.03
	PMSNP01379	1	2	0.42	-	-	0.03
	PMSNP01140	4	2	0.42	-	-	0.03
	PMSNP01126	4	2	0.43	-	-	0.03
	PMSNP01122	4	2	0.46	-	-	0.03
	PMSNP01461	6	3	0.73	-	0.42	0.10
Vein Density	PMSNP00271	5	2	0.073	-	-	0.03
	PMSNP00281	5	2	0.078	-	-	0.04
	PMSNP00285	5	2	0.076	-	-	0.03
	PMSNP00288	5	2	0.076	-	-	0.04
	PMSNP00289	5	2	-0.074	-	-	0.03

Note: For a multiallelic intercross QTL, there are two additive genetic effects.

Growth component traits, like leaf length, leaf width and leaf area, were observed to involve larger genetic components explained by QTLs (Table 1; Figure 3). Four QTLs contribute jointly to 20% of the phenotypic variation for leaf length, whereas over a half of the phenotypic variation for leaf width is explained by five QTLs. For leaf area, two QTLs detected account for 15% of its phenotypic variation. One QTL associated with marker PMSNP00307 on linkage group 5 pleiotropically affect both leaf length and width. Two multiallelic intercross QTLs also on linkage group 5 are pleiotropic QTLs for leaf width and area. Although these two traits are controlled by intercross QTLs, the dominant effects are relatively small, compared with their additive effects. A total of three QTLs explain about 9% of the phenotypic variation for leaf petiole.

Relative to growth traits, we found more QTLs involved in form traits; for example, five for stem taper and six for leaf shape (Table 1; Figure 2 and 3). A total of 15% and 23% of the phenotypic variation are due to these QTLs for the two traits, respectively. The number of veins per leaf is controlled by multiple QTLs from different linkage groups 1, 4, 5, and 6, totally explaining 20% of its phenotypic variation (Table 1; Figure 4). Five QTLs, all on linkage group 5, were detected to affect the density of veins, with 17% of the phenotypic variation explained.

## Discussion

Genetic mapping has proven to be a powerful tool in studying the genetic architecture and complex traits and designing marker-assisted selection programs for many species. Genetic linkage maps are generally constructed using a segregating progeny, such as the backcross, F_2_, or recombinant inbred lines, derived from two homozygous inbred lines. For perennial trees, however, it is difficult or impossible to obtain such inbred lines owing to their long-generation interval, high heterozygosity, and high inbreeding depression [15]. On the other hand, because of their high heterozygosity, the F_1 _full-sib family produced by crossing two trees may provide an adequate amount of information for linkage analysis [16]. In such a family, there are many types of segregating markers. Earlier pseudo-test backcross designs by Grattapalia and Sederoff [16,17] can make use of markers that are heterozygous in one parent but homozygous in the other, taking advantage of linkage analysis models developed for the backcross population. Since a more sophisticated model for linkage mapping has been developed [11-13,18], any type of markers segregating in a full-sib family can be analyzed by simultaneously estimating the linkage and linkage phases. This method was successfully used in poplar tree [14], sugarcane [19,20], a yellow passion fruit population [21], rubber tree [22] and peach [23].

This is a first study for mapping QTLs that control botanical traits in mei. By crossing two mei cultivars, a full-sib family was generated as a mapping population. Zhang et al.'s [3] sequencing result provides sufficient information for genotyping the mei genome. The high-density linkage map constructed by SSR markers, SNP markers and InDels [7] allows mei QTLs to be identified and estimated. In this mapping population, dramatic differences at phenotypic and genetic levels were observed in growth-related traits and botanical form traits in mei. Although the linkage map used covers a large portion of the mei genome, we did not identify many QTLs for stem growth traits. This may be due to two reasons. First, the mei trees are in their early stage of establishment in the field, when environmental perturbations are a major factor to affect tree growth. As trees are established, genes play an increasing role in growth and growth component traits. Such a transition pattern of genetic control after the establishment was observed in an experimental plantation of poplar hybrids [24,25]. Second, growth is a complex trait that is likely to involve a complex network of genetic interactions. We expect that epistasis due to different genes which main effects are not significant may contribute to the phenotypic variation of stem growth traits. A more powerful model that can analyze and estimate the genetic effects of all markers at the same time is crucial to confirm this speculation [26].

Although growth and its components, such as ones related to leaf size, have been mapped in many woody plants, QTL mapping of several important botanical traits, like stem taper, leaf shape and leaf anatomy, has received little attention. To our best knowledge, this is the first study aimed to map QTLs that control the number and density of veins. As physiological pipelines that transport water, nutrients and hormones, leaf veins have been thought to be associated with plant growth and adaptability [27]. The vein QTLs identified from this study will help to understand the genetic variation of leaf venation. Wu et al. [28] presented one of the first studies that map QTLs for leaf shape in forest trees, and found different patterns of action of QTLs on leaf size and leaf shape. In Wu's [19] study, QTLs for stem form were found in juvenile poplar trees. Given its ornamental value, botanical form traits in mei are part of breeding objectives. This study has for the first time reported on the detection of QTLs that control stem shape and leaf shape, providing useful information for marker-assisted selection of good-shaped mei cultivars. It is noted that different genomic regions control growth and shape, suggesting different genetic machineries that generate the phenotypic variation of these two types of traits.

We detected the pleiotropic control of the same QTL over two allometrically related traits, leaf length and leaf width. Similar pleiotropic QTLs were also observed for leaf width and leaf area. All these findings are of significant value to unveil the genetic basis of morphological and developmental integration as a mechanism for plants to adapt to environmental changes. Our data was about juvenile treesm, in which there is limitation to make a strong inference about developmental mechanisms. Yet, the current result from young trees, plus those from subsequent years, will enable us to link genes and development into a platform of interplay at which we are in a better position to chart the genotype-phenotype map through developmental trajectories [30].

## Materials and methods

### F_1 _hybrids and DNA extraction

Two mei cultivars, *P. mume *'Fenban' (BJFU1210120013) and *P. mume *'Kouzi Yudie' (BJFU1210120022), were selected from the Qingdao Mei Garden, Qingdao, China (36°04′N, 120°20′E), differing in many growth and morphological traits. The cross of the two cultivars generated a segregating F_1 _population, of which 190 seedlings (Voucher specimen accession number: BJFU1210120025-0214) were grown in the Xiao Tangshan Horticultural Trial, Beijing, China (40°02′N, 115°50′E). Total DNA was extracted from fresh young leaves of each seedling according to the plant genomic DNA extraction Kit (TIANGEN, Beijing, China) following the manufacturer's instructions.

### Marker genotyping and map construction

Sun et al. [7] described a procedure of identifying and genotyping SSR markers for the F_1 _hybrids of mei, including SSR primer design and screening and PCR amplification. A total of 144 multiallelic intercross markers were genotyped for the F_1 _hybrid population. The description of the procedure to identify SNP markers and InDels for mei was shown in Sun et al. [8]. To the end, 105 multiallelic intercross markers, 395 biallelic intercross markers and 1004 testcross markers segregating in the hybrids were generated.

Sun et al. [7] used JoinMap version 4 [18] to construct a genetic linkage map from SSR markers. The estimated recombination fractions between markers were converted to map distance in centiMorgan using the Kosambi map function. The map is composed of eight linkage groups paralleling to the haploid chromosome number of the mei genome. The total length of the map is 670 cM, with an average marker distance of 5 cM. The positions of SNPs, InDels and SSRs were identified as CDS, intron, 5′UTR, 3′UTR and intergenic regions according to mei genome GFF files. Thus, relative positions of SNPs and InDels on the SSR linkage map can be determined.

### Phenotypic measurements

During the fast-growing season of mei, usually in July or August, we measured leaf size and morphology for each F_1 _seedling. Three representative leaves chosen for phenotyping from the same tree are those located at the main stem with leaf plastochron index of 10 to 12. For each chosen leaf, leafblade length and width were measured, from which leaf areas were calculated. The number of stomata was counted for each leaf. At the end of the first growing season in the field, each seedling was evaluated for its main stem height and stem base diameter. Growth and its component traits used for QTL mapping are the height (HT) and diameter (DIA) of the main stem, leaf length (LL), leaf width (LW) and leaf area (LA). The botanical form traits of mei were derived from measured traits, including stem shape (measured by the ratio of DIA to HT) and leaf shape (measured by the ratio of LW to LL). Also, the density of veins on the leaf was calculated as the ratio of veins to leaf area. The averages of trait values over three measured leaves were used for QTL analysis.

### QTL identification

Since a high-density map was used for genetic mapping, we directly associated marker genotypes with phenotypic traits to detect significant QTLs using a likelihood approach. In this particular full-sib family, there are multiple marker types, testcross, biallelic intercross and multiallelic intercross. Here, we describe the model to analyze the genetic effects of a multiallelic intercross QTL [31,32]. Assume two alleles P_1 _and P_2 _for parent **P **and two alleles Q_1 _and Q_2 _for parent **Q**, which generate four progeny genotypes, along with genotypic values (μ_11_, μμ_12_, μ_21_, μ_22_), expressed as

$$\begin{array}{cc} {\text{P}}_{\text{1}}{\text{Q}}_{\text{1}}: & \mu_{\text{11}}=\mu +{\text{a}}_{\text{1}}+{\text{a}}_{\text{2}}+\text{d}\\ {\text{P}}_{\text{1}}{\text{Q}}_{\text{2}}: & \mu_{\text{12}}=\mu +{\text{a}}_{\text{1}}-{\text{a}}_{\text{2}}-\text{d}\\ {\text{P}}_{\text{2}}{\text{Q}}_{\text{1}}: & \mu_{\text{21}}=\mu -{\text{a}}_{\text{1}}+{\text{a}}_{\text{2}}-\text{d}\\ {\text{P}}_{\text{2}}{\text{Q}}_{\text{2}}: & \mu_{\text{22}}=\mu -{\text{a}}_{\text{1}}-{\text{a}}_{\text{2}}-\text{d} \end{array}$$

where μ is the overall mean, a_1 _is the allelic (additive) effect contributed by parent **P**, a_2 _is the allelic effect contributed by parent **Q**, and d is the dominant effect due to the interaction between alleles from the two different parents. The quantitative genetic analysis of testcross QTLs and biallelic intercross QTLs have been available in previous studies [17,28,29]. Biallelic intercross QTLs generate three genotypes in the progeny, allowing one additive effect (a) and one dominant effect (d) to be estimated. For testcross QTLs with two progeny genotypes, only one additive effect (a) can be estimated. In each case, the proportion of the total phenotypic variance explained by each QTL was calculated.

The significance of QTLs was tested by calculating the log-ratio of likelihoods under the null hypothesis (there is no QTL) and alternative hypothesis (there is a QTL) and comparing it with the chi-square distribution. When multiple SNPs were included for QTL mapping, the significance of SNP needs to be adjusted using the false positive rate (FDR) approach. The genome-wide threshold of significance was determined after the FDR adjustment.

### Competing interests

The authors declared that they have no competing interests.

### Authors' contributions

Conceived and designed the experiments: QZ. Performed the experiments: LS. Analyzed the data: YW CZ XL. Contributed reagents/materials/analysis tools: LS XY TC KM WY HP JW QZ. Wrote the paper: LS RW.

## Funding

Publication of this work is supported by grants from the Ministry of Science and Technology (2011AA100207, 2013AA102607), the State Forestry Administration of China (201004012), the Fundamental Research Funds for the Central Universities (NO.BLX2013011), and "One-thousand Person Plan" Award.

## Notes

This article has been published as part of *BMC Genetics *Volume 15 Supplement 1, 2014: Selected articles from the International Symposium on Quantitative Genetics and Genomics of Woody Plants. The full contents of the supplement are available online at http://www.biomedcentral.com/bmcgenet/supplements/15/S1.
